# Exploring Metal–Metal Composites: A Study on AA5083 Matrix-Cr Particles Reinforced Composites

**DOI:** 10.3390/ma17246246

**Published:** 2024-12-20

**Authors:** Serdar Özkaya

**Affiliations:** Faculty of Engineering, Metallurgical and Materials Engineering, Karadeniz Technical University, 61080 Trabzon, Turkey; sozkaya@ktu.edu.tr

**Keywords:** powder metallurgy, metal matrix composites, milling, AA5083, chromium

## Abstract

The objective of this study is to develop chromium-reinforced metal–metal composites utilizing an AA5083 aluminum alloy matrix through powder metallurgy while also examining their properties. Samples were produced by incorporating varying quantities of chromium (5%, 10%, and 15% by weight) into the AA5083 matrix. In order to ensure a uniform distribution of chromium particles, the powders were blended in a ball mill and subsequently hot-pressed at 500 °C under 500 MPa for a period of two hours in an argon atmosphere. The resulting samples were subjected to analysis in order to determine the effect of chromium content on the composites, with particular attention being paid to their microstructure, hardness, density, tensile properties, tribological performance and corrosion resistance. The findings demonstrated that an elevated chromium concentration markedly augmented the hardness of the composite, exhibiting a 50% enhancement in the 15 wt.% Cr composite. A 30% reduction in wear loss was observed for the same sample. The A10 sample (10 wt.% Cr) exhibited the greatest corrosion resistance, although this declined in the A15 sample due to increased porosity. Tensile strength increased by up to 10 wt.% Cr before decreasing at 15 wt.% Cr, which was also attributed to porosity. These findings demonstrate that chromium reinforcement enhances the mechanical and tribological performance of AA5083 composites, rendering them suitable for applications requiring high hardness and wear resistance.

## 1. Introduction

Over the years, aluminum Matrix Composites (AMCs) have garnered significant attention in the fields of engineering and technology due to their remarkable properties, such as being lightweight and high-strength, with exceptional wear resistance and excellent thermal conductivity [1,2,3,4,5]. These characteristics make AMCs highly suitable for a wide range of practical applications. For instance, AMCs are extensively used in the aerospace and automotive industries for components such as engine parts, brake rotors, and structural elements, where reduced weight and improved performance are critical. They are also employed in the electronics sector for heat sinks and packaging materials due to their excellent thermal conductivity. Additionally, AMCs have found applications in the defense and marine industries, where materials with high strength-to-weight ratios and corrosion resistance are vital [6,7]. AMCs are produced by combining the properties of a base alloy with the desired characteristics of reinforcement particles to create materials with enhanced performance. Among the numerous aluminum alloys available, AA5083 stands out as a highly suitable material for aerospace and automotive applications due to its exceptional properties, including its high strength, stiffness, and wear resistance [8,9,10]. Additionally, with a magnesium content of approximately 4%, AA5083 exhibits excellent corrosion resistance, making it an ideal choice for shipbuilding and pressure vessel applications [11,12]. Various types of particulate reinforcements, such as SiC, Al_2_O_3_, B_4_C, Gr, and TiB_2_, have been extensively studied and employed to improve the properties of AMCs [1,13,14,15]. However, the use of ceramic reinforcements introduces challenges, including poor wetting between the reinforcement and the matrix phase, resulting in weak interfacial bonding. This limitation hinders the material from achieving its desired strength and ductility [16,17,18]. Additionally, ceramic particles often agglomerate and detach from the matrix, further degrading the composite’s performance. These issues have limited the potential of ceramic-reinforced AMCs, prompting the exploration of alternative reinforcement strategies. One promising approach to overcoming these challenges is the development of metal–metal composites, where both the matrix and the reinforcement phases are metallic. Metal–metal composites offer strong interfacial bonding due to the formation of intermetallic compounds and other metallurgical reactions at the matrix-reinforcement interface. This strong bonding significantly enhances the mechanical and tribological properties of the composite. Materials such as Cu, Ni, Cr, and 316L stainless steel have been investigated as reinforcements in metal–metal composites [19,20]. Among these, chromium stands out for its ability to improve mechanical properties, corrosion resistance, and wear behavior. The influence of chromium extends beyond its ability to form carbides. When dissolved in the matrix, chromium enhances interfacial bonding through the formation of intermetallics, while undissolved chromium contributes to dispersion strengthening via chromium carbide formation [21,22]. The role of chromium in materials science is multifaceted and depends on its form and concentration. Within an optimal range, chromium carbides can enhance hardness and strength, contributing positively to the composite’s performance. However, excessive chromium carbide formation can lead to brittleness, compromising the material’s overall quality. Thus, determining the appropriate amount of chromium is crucial to achieving the desired properties for specific applications.

While numerous studies have investigated the addition of ceramic reinforcements to AA5083 matrix materials [23,24,25,26,27,28], research on AA5083-based metal–metal composites remains scarce. This gap presents an opportunity to explore the potential of chromium as a metallic reinforcement for AA5083 composites. The objective of this study is to produce AA5083/Cr metal–metal composites using the mechanical alloying method and to evaluate the influence of varying chromium content on the mechanical, tribological, and corrosion characteristics of the resulting composite material. By addressing the challenges associated with ceramic reinforcements, this study aims to contribute to the advancement of AMC technology and expand the application potential of AA5083-based composites.

## 2. Materials and Methods

In this study, AA5083-Cr metal–metal composites were produced using the mechanical alloying method, which represents an advanced stage of the powder metallurgy technique. The mean particle size of AA5083 was 63 µm, while that of the chromium powder was 27 µm. The effect of chromium reinforcement content on the mechanical, tribological, and corrosion properties of the composite was examined. AA5083 powders, with the chemical composition shown in Table 1, were used as the matrix material.

Firstly, composite powders containing 5%, 10% and 15% by weight of Cr were prepared and mechanically alloyed in high-energy mills for 5 h. The milling process was carried out with a ball-to-powder ratio of 1:10 and a milling speed of 400 rpm. To prevent cold welding and minimize particle agglomeration, 3 wt.% methanol was added to the milling bowls as a process control agent (PCA). These optimized parameters ensured a uniform distribution of Cr particles within the AA5083 matrix, contributing to the improved microstructural and mechanical properties of the resulting composite material These samples were then coded as A0 (unreinforced), A5, A10 and A15, respectively. After the mechanical alloying process, the powders were hot-pressed under a pressure of 500 MPa at 500 °C for 2 h in a protective argon gas atmosphere. Following metallographic preparation, the samples were analyzed for density/porosity, microstructure, hardness and tensile strength. Density measurements were performed using the Archimed’s method, while porosity was determined using the following equation:

(1) $$Porosity\left(\%\right)=\frac{Teoritical\;density-Experimental\;Density}{Teoritical\;density}\;\times \;100$$

The microstructural investigations were conducted using a ZEISS Evo LS10 model (ZEISS, Jena, Germany) scanning electron microscope, while the phase analysis of the composites was conducted by X-ray diffraction using PANalytical/X’Pert^3^ Pro (Bruker, Westbrough, MA, USA) under Cu-Kα radiation (1.541874 Å) at 45 kV and 40 mA. XRD patterns were recorded in the 2θ range of 30–90° (step size = 0.01° and time step = 1 s). The Brinell hardness values of the metallographically prepared samples were measured using an Innovatest-Nemesis 9000 model testing device (Innovatest, Maastricht, The Netherland) in accordance with a load of 31.25 kg and a 2.5 mm diameter indenter. Tensile strength tests were conducted using an MTS Criterion Universal Testing Machine (MTS, Eden Prairie, MN, USA) at a tensile speed of 1 mm/min. The wear properties of the samples were investigated using the ball-on-disk wear test method under a 10 N normal load and a sliding speed of 0.471 m/s over a sliding distance of 100 m by using a 6 mm diameter abrasive tungsten carbide ball. Potentiodynamic polarization tests were conducted to evaluate the effect of Cr reinforcement on corrosion properties. These tests were performed using a Gamry Reference 3000 corrosion testing device (Gamry Instruments, Warminster, PA, USA). The potentiodynamic polarization tests were conducted using a three-electrode setup in a 3.5 wt.% NaCl aqueous solution at ambient temperature. The AA50834/Cr MMC samples, with dimensions of 5 mm × 5 mm × 10 mm, served as the working electrode. Electrical connections were established by attaching the samples to copper wires, and they were embedded in resin for support. A graphite rod was used as the counter electrode, while an Ag/AgCl (SCE) electrode functioned as the reference electrode. To allow the open-circuit potential to stabilize, a 30 min delay was applied before initiating the test. The corrosion behavior of the samples was assessed by determining the corrosion potential (*Ecorr*) and corrosion current density (*Icorr*) from the potentiodynamic polarization curves and analyzed using the Tafel extrapolation method. The experiments were conducted at a scan rate of 1 mV/s over a voltage range of −1 V to +0.5 V. Each test was repeated a minimum of three times to ensure consistent and reliable results.

## 3. Results

### 3.1. Hardness and Porosity

**Figure 1:**
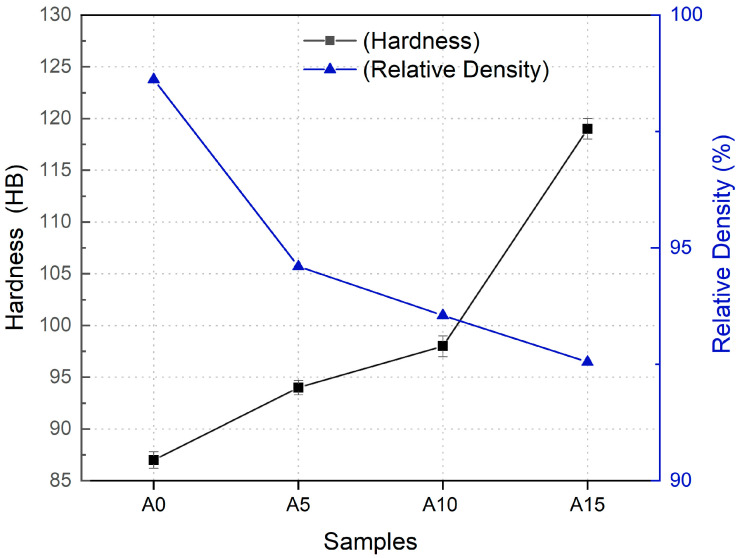
Relative density and hardness values of AMCs containing different amounts of Cr.

Figure 1 presents the hardness and relative density graphs of AA5083-Cr composite samples. The hardness values show a consistent increase with rising Cr content, achieving an approximate 50% improvement from sample A0 to sample A15. This increase can be attributed to multiple factors related to the reinforcement of Cr particles in the AA5083 matrix through the powder metallurgy process. One primary factor is solid solution strengthening. Chromium atoms partially dissolve in the aluminum matrix, disrupting the crystal lattice and hindering dislocation motion, thereby enhancing hardness [29]. Additionally, the dispersion of chromium particles within the aluminum matrix creates a second phase that obstructs dislocation movement, further contributing to the observed hardness increase [30]. The strong interfacial bonding between the chromium particles and the aluminum matrix improves the load-bearing capacity of the composite, amplifying its mechanical performance. The mechanical milling process also introduces deformation into the powders, enhancing hardness by refining the microstructure. Furthermore, chromium’s tendency to form precipitates within the aluminum matrix plays a pivotal role in the hardening mechanism. These precipitates act as obstacles to dislocation motion, thereby reinforcing the material’s hardness [31]. It is noteworthy that the dominant crystallographic plane observed in the AA5083/Cr composite samples is (111), a plane that is renowned for its high atomic packing density and resistance to deformation. The prevalence of this plane correlates with the observed increase in hardness, as the (111) plane in face-centered cubic (FCC) structures like aluminum offers higher resistance to dislocation motion compared to other crystallographic planes [32]. This reinforces the mechanical performance of the composite by serving as a naturally hard plane, which synergizes with the strengthening effects of the Cr particles. Conversely, the relative density curve reveals a decline with increasing Cr content, indicating higher porosity levels in the samples. At first glance, this appears contradictory since porosity typically correlates inversely with hardness and increased porosity often reduces hardness. However, this paradox is resolved when considering the significantly higher hardness of the Cr reinforcement material (125 HB) compared to the AA5083 matrix (85 HB). The superior hardness of Cr compensates for the negative impact of increased porosity, ultimately resulting in a net increase in hardness across the composite samples [19].

### 3.2. Microstructural Investigation and XRD Analyses

**Figure 2:**
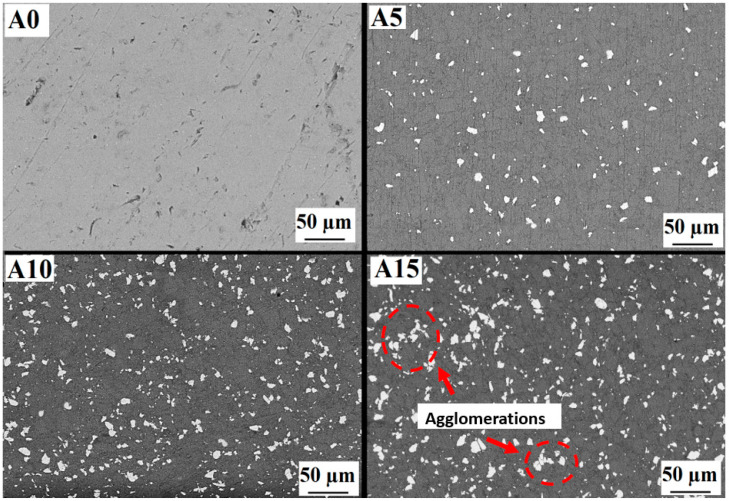
Microstructures of A0, A5, A10 and A15 samples.

Figure 2 shows the microstructures of AA5083/Cr composites with varying Cr particle content. In the images, the light-colored phases represent Cr particles and the dark regions correspond to the aluminum alloy matrix. These microstructure images allow for an analysis of Cr particle distribution within the matrix and the homogeneity of the composite structure. In specimens A5 and A10, Cr particles are observed to be distributed homogeneously throughout the matrix. This homogeneous dispersion is crucial for enhancing the composite’s mechanical properties, as it ensures consistent material performance across the structure [33,34]. The primary reason for this uniform distribution is the mechanical alloying process applied before pressing [35,36]. Mechanical alloying ensures thorough mixing of the powders, which promotes even distribution during subsequent processing. However, in sample A15, a localized agglomeration of Cr particles is observed. Agglomeration refers to the clustering and aggregation of Cr particles within the matrix. This phenomenon becomes more pronounced as the reinforcement content increases, leading to a loss of homogeneity. Such localized agglomerations can weaken certain areas of the composite, adversely affecting its overall mechanical properties. They may also act as stress concentrators, potentially reducing the material’s performance under load. The mechanical alloying process typically prevents agglomeration by promoting fusion and a uniform distribution of powders. However, as the reinforcement content surpasses a certain threshold, maintaining homogeneity becomes more challenging. This underscores the importance of optimizing the reinforcement content to achieve a balance between improved mechanical properties and maintaining structural uniformity.

**Figure 3:**
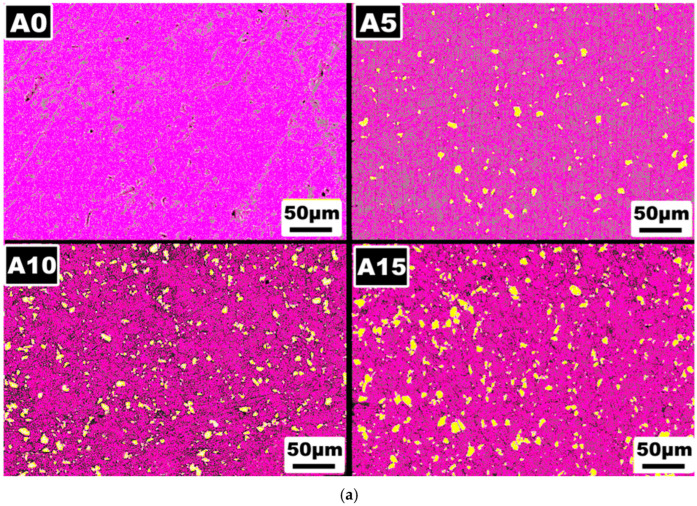

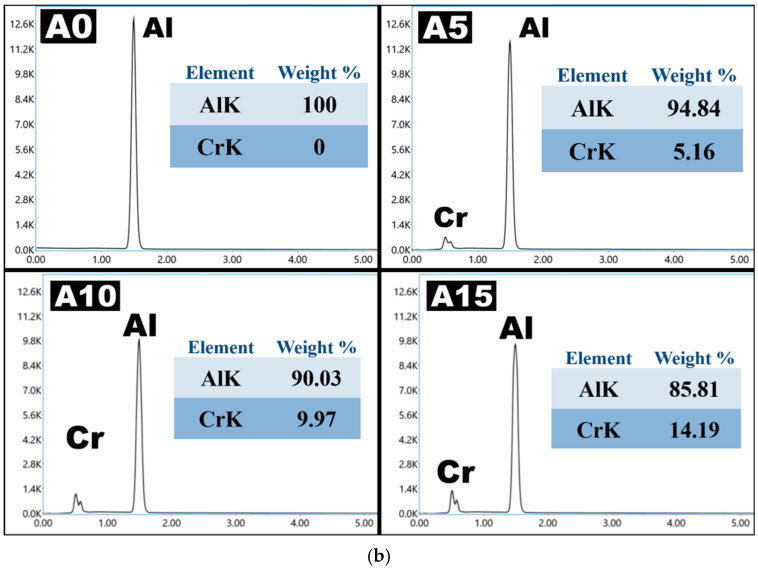
(**a**) Elemental mapping and (**b**) elemental analyses of samples.

The elemental mapping photograph obtained at high magnification for samples are presented in Figure 3a. In this image, the purple regions represent the AA5083 phase and the yellow regions indicate the Cr phase. As shown in the figure, there is a distinct separation between the purple and yellow areas. If an intermetallic phase had formed in the structure, regions with overlapping purple and yellow colors would be visible, indicating the presence of both elements in the same phase. However, the sharp boundary observed in the elemental mapping confirms that no intermetallic phase is present in the structure. A comparison can be drawn with a similar study in which AA2024/316L SS particle-reinforced composites were produced [37]. In that study, intermetallic phases formed at the interfaces between the matrix and reinforcement were visible as mixed regions in the elemental mapping, reflecting the presence of both elements. In contrast, in the current study, as verified by both the XRD analysis and elemental mapping results, no secondary phase or intermetallic compound formation is detected. This finding further emphasizes the distinct separation between the AA5083 and Cr phases in the composite. Moreover, elemental analyses of the samples were conducted to evaluate the distribution and composition of the matrix and reinforcement elements (Figure 3b). The results indicated that the ratio of the matrix element (Al) to the reinforcement element (Cr) does not exhibit any drastic changes. This consistency can be attributed to the uniform distribution of Cr particles achieved during the mechanical milling and consolidation processes. The addition of Cr reinforcement did not alter the matrix composition significantly, as the primary elements of the AA5083 alloy remained stable across all samples. This suggests that the powder metallurgy process, combined with optimized milling parameters and the use of a small amount of methanol to prevent agglomeration, successfully maintained the elemental integrity of the matrix while incorporating the reinforcement.

**Figure 4:**
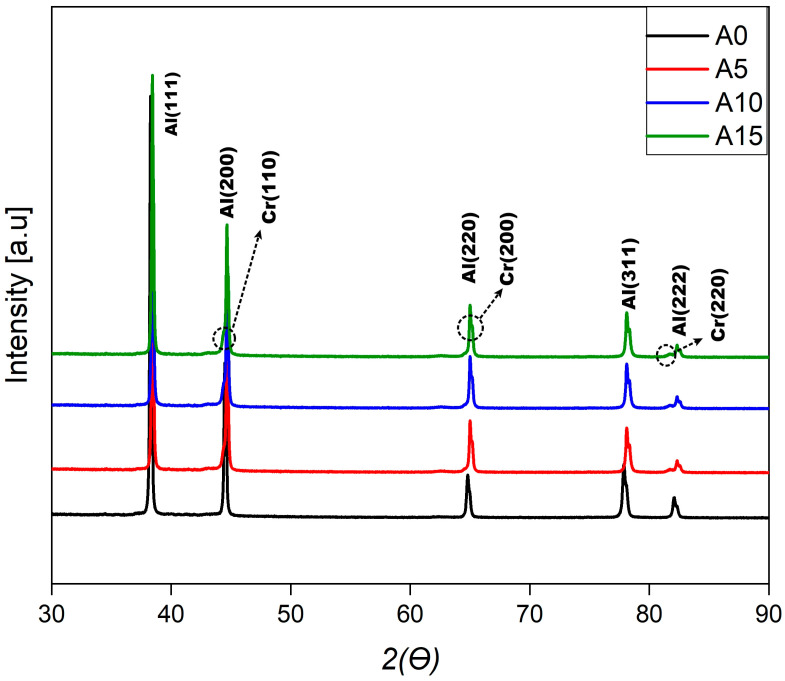
XRD patterns of samples.

The X-ray diffraction (XRD) spectra for the base metal and composite samples are shown in Figure 4. Analysis of the XRD spectra reveals the most prominent peak phases at 2θ values of 37.94°, 44.19° and 60.00°. These peaks correspond to the hkl Miller index planes (111), (200), (220), (311) and (222), respectively [38]. An increase in the quantity of Cr reinforcement leads to an enhancement in the intensity of the XRD peaks, which suggests an improvement in the crystallographic structure of the materials. The absence of intermetallic and oxide peaks in all samples indicates that the hot-pressing process was insufficient in regard to promoting oxidation and effectively prevented interactions between the AA5083 and Cr materials [38]. The solubility of chromium in aluminum is limited, and the formation of new intermetallic phases in the AA5083 alloy occurs only if this solubility limit is exceeded. In this study, it is likely that the chromium remained dissolved in the alloy without forming intermetallic phases due to its constrained solubility. Additionally, a structural mismatch between the crystal structure of the AA5083 alloy (face-centered cubic, FCC) and that of chromium (body-centered cubic, BCC) could impede the orderly incorporation of chromium atoms into the aluminum matrix. This incompatibility may hinder the formation of new intermetallic phases [14,39].

### 3.3. Tensile Strength and Elongation

**Figure 5:**
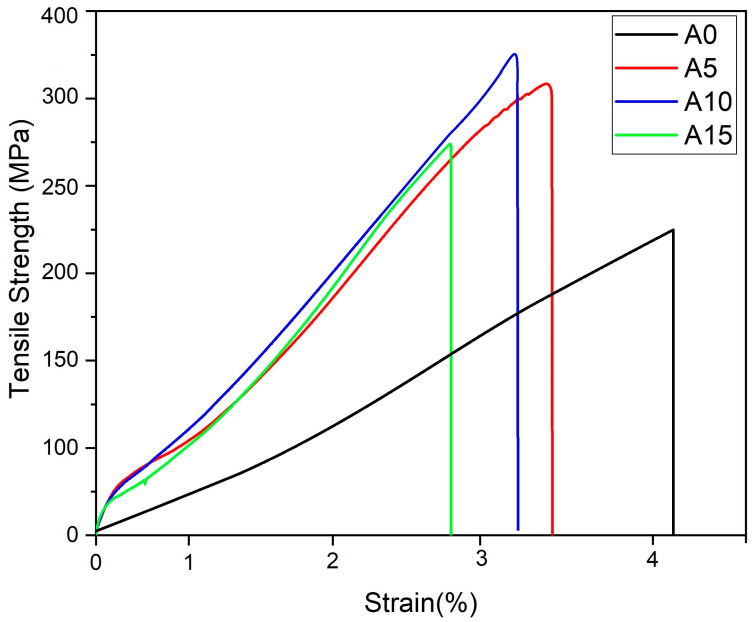
Tensile strength–strain plots of samples.

The tensile strength and elongation of Cr-reinforced AA5083 metal matrix composites were evaluated for varying Cr reinforcement ratios (0%, 5%, 10%, and 15% by weight), as illustrated in Figure 5. The results, which are also indicated in Table 2, reveal significant variations in tensile strength and elongation in correspondence with changes in Cr content. The unreinforced AA5083 sample exhibited the lowest tensile strength at 220 MPa. A remarkable 40% increase in tensile strength was observed in the A5 sample with 5% Cr reinforcement, reaching 304 MPa. This improvement is attributed to the uniform distribution of Cr particles within the matrix, which enhances the material’s load-carrying capacity. The tensile strength peaked at 324 MPa in the A10 sample, representing a 45% increase compared to the unreinforced material. This maximum strength is due to an optimal combination of Cr particle dispersion and interfacial bonding, which effectively transfers and distributes the applied stress. However, a decline in tensile strength was noted in the A15 sample despite its strength being higher than the unreinforced specimen. This reduction is attributed to the heterogeneous distribution of Cr particles at higher concentrations, which results in increased porosity [40,41]. Porosity introduces microstructural defects that compromise the mechanical integrity of the composite, counteracting the strengthening effect of Cr reinforcement. Conversely, elongation values decreased consistently with increasing Cr content. The unreinforced sample exhibited the highest elongation, reflecting its superior ductility. In contrast, the A5 and A10 samples showed significant reductions in elongation, with the lowest elongation being recorded in the A15 sample. This trend is due to the Cr particles restricting the free mobility of the aluminum matrix, thereby reducing the ductility of the composite [40,42]. Although Cr reinforcement enhances hardness, it also correlates with an increase in porosity. The elevated porosity in the A15 sample is a critical factor behind its reduced tensile strength relative to the A5 and A10 samples. These findings underscore the complex interplay between Cr reinforcement, porosity, and mechanical properties. Thus, the mechanical behavior of Cr-reinforced AA5083 composites is highly dependent on the reinforcement ratio. While moderate Cr reinforcement (5–10 wt.%) optimizes tensile strength, excessive Cr content (15 wt.%) leads to diminished performance due to microstructural inhomogeneities and increased porosity.

### 3.4. Wear Behavior

**Figure 6:**
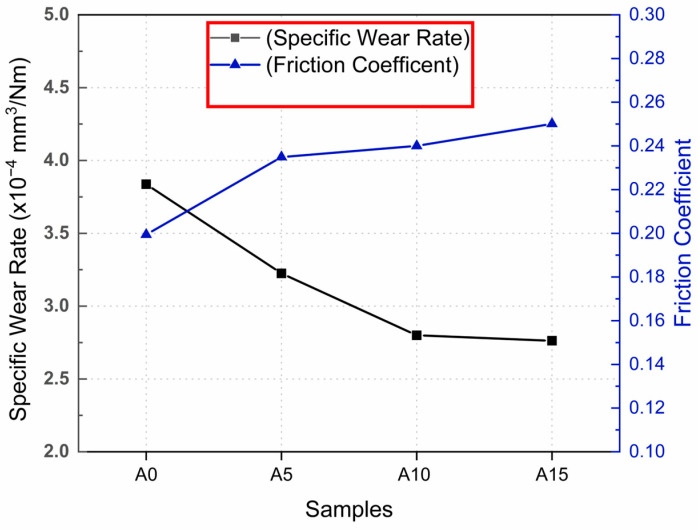
Specific wear loss and friction coefficient of samples changing with Cr content.

**Figure 7:**
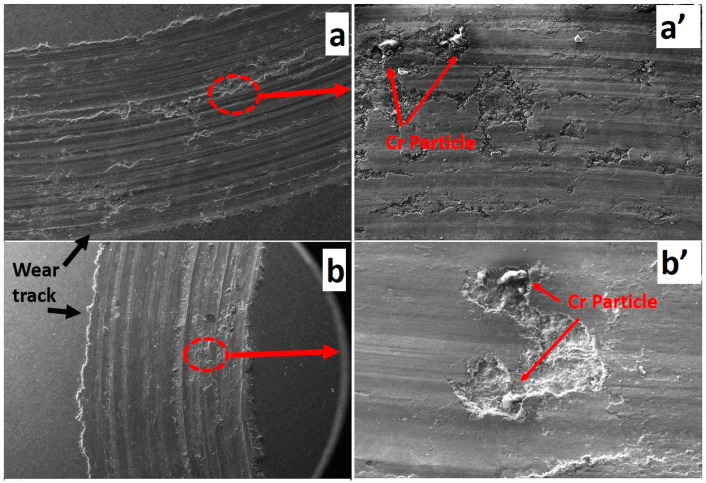
The 50× (**a**,**b**) and 1000× (**a’**,**b’**) magnificated worn surfaces of the A10 (**a**,**b**) and A15 (**a’**,**b’**) samples.

**Figure 8:**
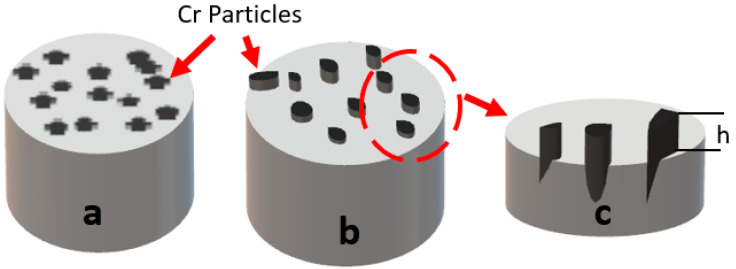
Figural drawing showing the effect of Cr particles on surface adhesion and wear: (**a**) smooth, (**b**) rough and (**c**) wear-resistant surfaces.

The specific wear rate and friction coefficient values of the tested samples are presented in Figure 6. The AA5083 matrix exhibited the highest wear loss among all tested materials, primarily due to its ductile nature. Being devoid of any reinforcement, the unmodified AA5083 matrix is more susceptible to deformation when subjected to the counter-abrasive ball during wear. The addition of Cr as a reinforcement significantly reduced the wear loss of the composites, with a continuous decrease being observed as the Cr particle concentration increased. For the A5 sample (5 wt.% Cr), wear loss was reduced by approximately 16% compared to the unreinforced AA5083 matrix. This reduction further improved in the A10 and A15 samples, with decreases of 25% and 30%, respectively. This enhanced wear resistance can be attributed to the Cr particles on the surface acting as barriers to abrasion, as illustrated in Figure 7a’,b’. When the abrasive ball moves across the composite surface, it must overcome the Cr particles before abrading the softer matrix phase [42,43,44,45]. Chromium (Cr) particles play a pivotal role in enhancing the wear resistance of AA5083/Cr composites. These particles form a robust barrier against abrasion, effectively shielding the softer aluminum matrix. Due to their high hardness and superior shear strength, Cr particles resist plastic deformation and prevent direct interaction between the abrasive counterbody and the matrix material. When subjected to wear, the Cr particles either endure abrasion or remain intact thanks to their strong adhesion to the matrix, which prevents detachment. Unlike ceramic-reinforced composites, where reinforcement particles often detach under stress, metal–metal composites benefit from excellent wetting and robust interfacial bonding, ensuring the retention of Cr particles during wear (Figure 8). This retention mechanism ensures that the abrasive ball interacts with the Cr particles, leveraging their hardness and high shear strength to minimize material loss [43]. The graphical representation of the friction coefficients reveals a positive correlation between the coefficient of friction and Cr concentration. Although this may initially seem counterintuitive alongside the observed reduction in wear loss, the explanation lies in the nature of the composite design. The increasing Cr content results in a harder surface, leading to greater frictional interactions during sliding [46]. However, this same hardness effectively prevents material removal, thereby reducing overall wear. In conclusion, Cr reinforcement enhances the wear resistance of AA5083 composites through the presence of hard, high-shear-strength Cr particles, robust interfacial bonding, and resistance to detachment [1,47]. While the coefficient of friction increases with higher Cr content, it does not compromise wear resistance, demonstrating the advantageous balance provided by the metal–metal composite design.

In the AA5083 composite, the reinforcement phase, which is firmly embedded in the matrix, is harder than the matrix phase. During the wear test, this phase cannot be suddenly detached from the surface, resulting in the formation of rough peaks, as illustrated in Figure 8. Additionally, the presence of Cr particles, which are harder and more rigid, makes them more difficult to abrade, contributing to an increase in the friction coefficient due to surface roughness. A combined examination of weight loss and friction coefficients confirms this mechanism. Despite the increase in the friction coefficient, a concomitant decrease in weight loss was observed, indicating that the Cr particles provided resistance to wear during the test and protected the matrix phase against the abrasive surface.

### 3.5. Corrosion Behavior

**Figure 9:**
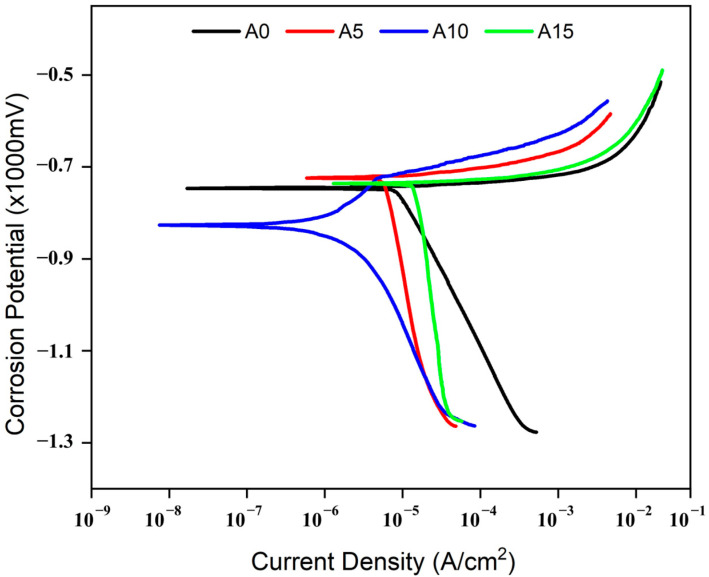
Potentiodynamic polarization profiles for both the composite materials and the AA5085 alloy.

The potentiodynamic polarization curves of the composites and the AA5083 alloy are illustrated in Figure 9. The AA5083 alloy (represented by the black curve) serves as a reference, demonstrating the electrochemical behavior of the unmodified alloy. This curve reveals a specific corrosion potential (*Ecorr)* and current density (*Icorr*) which reflect the inherent corrosion rate and susceptibility of the alloy. When 10% chromium (Cr) is added to the AA5083 alloy (depicted by the blue curve), there is a significant shift in the corrosion potential to more negative values and a reduction in the current density compared to the base alloy. This shift may suggest a slightly increased tendency for corrosion initiation due to the presence of chromium [48]. The AA5083 alloy with 5% Cr (illustrated by the red curve) exhibits a corrosion potential similar to that of the unmodified AA5083 alloy but has a higher current density. The similarity in *Ecorr* indicates that the corrosion resistance is comparable to pure AA5083; however, the increased *Icorr* signifies a more aggressive corrosion process once it begins. The AA5083 alloy with 15% Cr (shown by the green curve) displays the highest current density among the samples, indicating a greater likelihood of corrosion initiation. Despite the elevated current density, it is not the highest density observed, suggesting complex corrosion behavior that might involve mechanisms beyond the mere presence of chromium [4,30].

Table 3 summarizes the corrosion data for AA5083 samples reinforced with varying amounts of Cr particles, including unmodified AA5083 and AA5083 reinforced by 5%, 10% and 15% Cr. Corrosion potential (*Ecorr*) is reported in millivolts (mV), corrosion current density (*Icorr*) in microamperes per square centimeter (μA/cm^2^) and corrosion rate in mils per year (mpy). The data show that, while the *Icorr* values generally increase with the addition of Cr, the 10% Cr sample exhibits a significant improvement, with a nearly fourfold increase in corrosion resistance compared to unreinforced AA5083. This enhancement can be attributed to the ability of Cr to promote passivation, a process that reduces the material’s reactivity to environmental factors such as air and water. However, as Cr content increases beyond 10%, corrosion resistance declines notably, particularly in the 15% Cr sample. This decline is primarily due to increased porosity resulting from particle agglomeration and uneven Cr distribution in the aluminum matrix. Porosity provides pathways for corrosive agents like NaCl to penetrate the material, initiating localized corrosion [49]. Furthermore, the presence of Cr particles introduces potential differences within the matrix, forming microgalvanic cells that accelerate the anodic dissolution of the aluminum near Cr particles. These microgalvanic effects become more pronounced at higher Cr contents, compounding the loss of corrosion resistance [4,30]. Excessive Cr content also leads to the clustering of Cr particles, which weakens interfacial bonding between the matrix and reinforcement. This hinders the formation of a uniform, stable passive oxide layer—a crucial barrier against corrosion. The lack of a continuous oxide layer leaves the composite vulnerable to attack, undermining the benefits of Cr reinforcement. While moderate Cr content, such as 10%, effectively enhances corrosion resistance by promoting passivation, higher concentrations negate these benefits due to the combined effects of porosity, galvanic activity, and impaired oxide-film stability. These findings underscore the critical importance of optimizing Cr content to achieve a balance between mechanical performance and corrosion resistance in AA5083-Cr composites.

## 4. Conclusions

In this study, AA5083/Cr metal–metal composites were successfully fabricated using powder metallurgy, and their properties were investigated. It was demonstrated that a new type of composite, metal–metal composites, can be produced through the fabrication of AA5083-Cr composites. This innovative approach opened up possibilities for enhancing the properties of the base AA5083 alloy with the addition of chromium as a reinforcement phase. As the concentration of Cr increased, the hardness of the samples rose, accompanied by a decline in their relative density. This indicates that the addition of Cr particles contributes to a harder composite structure, although it also impacts the material’s overall density, suggesting a trade-off between hardness and density. No secondary phase formation was observed in the AA5083-Cr material, which implies that the chromium particles were uniformly distributed within the matrix without forming separate, identifiable phases. This uniform distribution may be beneficial in maintaining the integrity and consistency of the composite properties. The tensile strength of the composite increased with the amount of Cr reinforcement up to the A10 specimen. However, the A15 specimen exhibited lower tensile strength than both the A5 and A10 specimens, indicating a non-linear relationship between Cr concentration and tensile strength. Additionally, the percentage elongation of the samples diminished with the incorporation of Cr reinforcement, reflecting a reduction in ductility as the amount of reinforcement increased. The presence of Cr particles on the surface acted as a barrier against wear, leading to an increase in the friction coefficient and a reduction in wear loss as the Cr content rose. This suggests that chromium reinforcement enhances the wear resistance of the composite by providing a protective surface layer that resists abrasion. Finally, the sample containing 10 wt.% Cr demonstrated the highest corrosion resistance, while the sample containing 15 wt.% Cr showed the lowest resistance. This finding indicates that, while the addition of chromium can improve certain properties, excessive Cr content can lead to microstructural inhomogeneities that reduce the overall corrosion resistance of the material.

## Figures and Tables

**Table 1:** Chemical Composition of AA5083 [7].

Element	Si	Fe	Cu	Mn	Mg	Zn	Ti	Cr	Al
Composition (wt.%)	0.4	0.4	0.1	0.4–1.0	4.0–4.9	0.25	0.15	0.05–0.25	Balance

**Table 2:** Tensile strength and strain values of samples.

Sample	Tensile Strength (Mpa)	Strain (%)
A0	220	4.3
A5	304	3.2
A10	324	3.1
A15	276	2.8

**Table 3:** Parameters of electrochemical nature obtained from the polarization studies.

Sample	*Ecorr* (mV)	*Icorr* (μA)	Corrosion Rate (mpy)
A0	−746	6.94	13.46
A5	−723	8.86	17.6
A10	−827	1.85	3.59
A15	−737	23.10	34.43

## Data Availability

The original contributions presented in this study are included in the article. Further inquiries can be directed to the author.
